# Deep learning‐based prediction of H3K27M alteration in diffuse midline gliomas based on whole‐brain MRI

**DOI:** 10.1002/cam4.6363

**Published:** 2023-07-17

**Authors:** Bowen Huang, Yuekang Zhang, Qing Mao, Yan Ju, Yanhui Liu, Zhengzheng Su, Yinjie Lei, Yanming Ren

**Affiliations:** ^1^ Department of Neurosurgery West China Hospital of Sichuan University Chengdu China; ^2^ Department of Pathology West China Hospital of Sichuan University Chengdu China; ^3^ College of Electronics and Information Engineering Sichuan University Chengdu China

**Keywords:** convolutional neural network, diffuse midline gliomas, H3K27M alteration, radiomics, transformer

## Abstract

**Background:**

H3K27M mutation status significantly affects the prognosis of patients with diffuse midline gliomas (DMGs), but this tumor presents a high risk of pathological acquisition. We aimed to construct a fully automated model for predicting the H3K27M alteration status of DMGs based on deep learning using whole‐brain MRI.

**Methods:**

DMG patients from West China Hospital of Sichuan University (WCHSU; *n* = 200) and Chengdu Shangjin Nanfu Hospital (CSNH; *n* = 35) who met the inclusion and exclusion criteria from February 2016 to April 2022 were enrolled as the training and external test sets, respectively. To adapt the model to the human head MRI scene, we use normal human head MR images to pretrain the model. The classification and tumor segmentation tasks are naturally related, so we conducted cotraining for the two tasks to enable information interaction between them and improve the accuracy of the classification task.

**Results:**

The average classification accuracies of our model on the training and external test sets was 90.5% and 85.1%, respectively. Ablation experiments showed that pretraining and cotraining could improve the prediction accuracy and generalization performance of the model. In the training and external test sets, the average areas under the receiver operating characteristic curve (AUROCs) were 94.18% and 87.64%, and the average areas under the precision‐recall curve (AUPRC) were 93.26% and 85.4%.

**Conclusions:**

The developed model achieved excellent performance in predicting the H3K27M alteration status in DMGs, and its good reproducibility and generalization were verified in the external dataset.

## 1 INTRODUCTION

H3K27M‐mutant diffuse midline glioma (H3K27M‐DMG) is a new class in the 2016 WHO classification of central nervous system (CNS) tumors that combines gene mutation and histopathological features.^1^ H3K27M mutation was replaced by H3K27M alteration in the 2021 WHO classification of CNS tumors (5th edition).^2^ Regardless of its histological features, a tumor in this class has a WHO grade of IV and can appear in patients of all ages.^3^, ^4^, ^5^ In both adult and pediatric patients with diffuse midline gliomas (DMGs), the H3K27M‐altered type is an independent factor that results in worse overall survival (OS) than wild‐type H3K27M.^6^, ^7^, ^8^, ^9^

Due to the invasive growth patterns of these tumors, their deep growth locations, and the surrounding important functional areas, the effect of surgery or radiotherapy and chemotherapy on the prognosis of patients is very limited.^10^, ^11^, ^12^, ^13^, ^14^ Currently, new therapeutic targets and targeted drugs for H3K27M‐altered tumors have been identified and developed, which may lead to effective treatment for patients in the future.^15^, ^16^ Early diagnosis enables early treatment. It has been reported that whether H3K27M is altered can be judged by cerebrospinal fluid examination, but the detection rate is only 10%.^17^ At present, tissue biopsy is the main method to detect H3K27M alterations, but it often requires craniotomy, which is an invasive and high‐risk inspection method. A survey shows that more than half of surgeons (59%) will not consider routine biopsy for DMG.^18^

Magnetic resonance imaging (MRI) is a noninvasive examination that can provide images of the whole tumor, offering advantages over stereotactic or open biopsy which provide only limited amounts of tumor tissue for molecular diagnoses and may be subject to sampling bias. However, the tumor appearance observed by the naked eye on MRI shows diversity, and its enhancement features, border features, infiltration appearance, and oedema are not enough to distinguish H3K27M alterations.^19^, ^20^ In recent years, convolutional neural networks (CNNs) in depth learning have been increasingly used in the field of medical imaging, and deep learning has been shown to outperform previous state‐of‐the‐art machine learning techniques in multiple domains.^21^, ^22^

The objective of this study was to construct an automated prediction model for H3K27M alterations in DMG based on deep learning and whole‐brain MRI. It is hoped that this model will help patients obtain an early and rapid diagnosis, and provide a new noninvasive alternative detection method for patients who do not want to undergo surgical biopsy.

## 2 MATERIALS AND METHODS

### 2.1 Patients

The flowchart of the study population is shown in Figure S1. In this retrospective study, patients who underwent tumor resection or biopsy from February 2016 to April 2022 were recruited from West China Hospital of Sichuan University (WCHSU) and Chengdu Shangjin Nanfu Hospital (CSNH). All patients were newly diagnosed with DMG with H3K27M status detected. The exclusion criteria were as follows: (1) history of preoperative surgery or radiation and chemotherapy; (2) lack of preoperative contrast‐enhanced T1‐weighted (T1C) or T2 sequences; and (3) artifact interference on preoperative MRI. H3K27M status was determined by pyrosequencing analysis for H3F3A or HIST1H3B mutations. T1C and T2 sequences were collected from preoperative MRI for our study, as the features of these two sequences are the most effective in terms of distinguishing H3K27M mutation status.^23^, ^24^, ^25^ From the public IXI dataset (https://brain‐development.org/), a total of 75,365 brain MR images of approximately 600 healthy subjects were collected for model pretraining. This study was conducted in accordance with the principles of the Declaration of Helsinki.

### 2.2 MRI data acquisition and tumor segmentation

At WCHSU, MRI was performed on 3.0‐T MR scanners (Philips Achieva; GE MR 750 W; Siemens Healthcare) or 1.5‐T clinical scanners (Toshiba Medical Systems; Alltech Medical Systems). The major MRI protocol included the following sequences: axial T1C (repetition time [TR]: 1550 ms, echo time [TE]: 1.98 ms, slice thickness [ST]: 5–6 mm) and axial T2 sequences (TR: 4500 ms, TE: 105 ms, ST: 5–6 mm). At CSNH, MRI was performed on 3.0‐T (uMR780 3.0 T, UIH) or 1.5‐T (Achieva 1.5 T, Philips Medical Systems) MR scanners. The major MRI protocol included the following sequences: T1C (TR\TE: 151.8\2.40 ms, ST: 5–6 mm) and T2 axial sequences (TR\TE: 3943\100 ms, ST: 5.5–6 mm). Detailed MRI equipment information and scanning schemes are described in Table S1. The 2D regions of interest (ROI) of each image included the tumor enhancement region in the T1C sequence and the tumor edema region in the T2 sequence. Manual slice‐by‐slice segmentation was performed on the axial plane by a neurosurgeon with 5 years (B.H.) of experience using LabelMe software version 3.16.2 and later revised by a neurosurgeon with 10 years of clinical experience (Y.R.). Some examples of tumor segmentation are shown in Figure S2.

### 2.3 Study design

**FIGURE 1:**
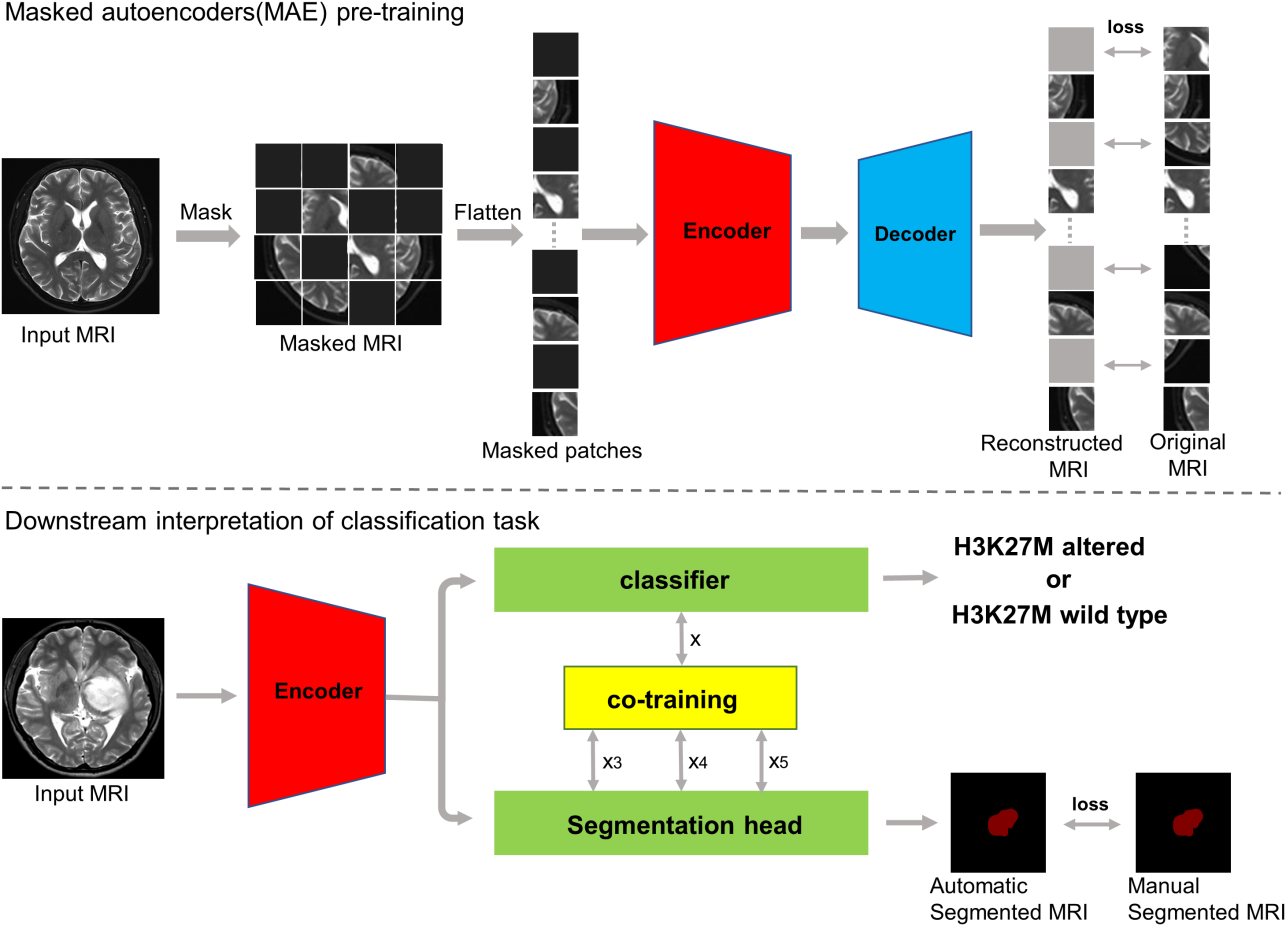
Illustration of the entire model structure and training process. The top part is MAE pretraining. The lower part of this figure represents the downstream classification task.

The structure diagram of our model is shown in Figure 1. The training of our model is divided into two parts. In the first part, we use Masked AutoEncoder (MAE) to pre train the encoder part of the model. In the second part, we use the cotraining method to train our own tagged data with specific classification tasks and fine tune the encoder parameters after pretraining in the first part. MAE is a new generative self‐monitoring learning model proposed by He Kaiming^26^ in 2021. It has an asymmetric encoder‐decoder structure, which can automatically reconstruct the original image with a small number of patches, increasing the adaptability of the model to tasks in the same scene.

At present, CNN is widely used in the field of computer vision, the convolutional operation only works with local neighborhoods, and it is effective in extracting local features. It is difficult to capture interactive features over long distances, which affects the accuracy of classification to some extent.^27^ Therefore, we introduced a transformer. Compared with a CNN, it achieves a significant performance improvement in global feature extraction via a self‐attention mechanism.^28^

We added a branch task—tumor automatic segmentation task—to the main task (classification task) and adopted cotraining. As segmentation and classification are two related tasks, they can be trained at the same time, and the features in the classification head and segmentation head can be transferred to each other. In the process of transmission, the attention mechanism is used to focus on the features that can improve the accuracy of model classification.

### 2.4 Development of deep learning algorithms

The detailed overall structure of our baseline model is described in Figure S3, where the detailed structure of the transformer, resblock 1, and resblock 2 modules is shown in Figure S4. First, T2 images of the healthy head were used to pretrain the encoder part of the model. In the pretraining, we used an Adam optimizer with a momentum of 0.9 and a weight decay of 1e‐4, the initial learning rate was 0.05, and the number of epochs was 1000. The loss function computed the mean squared error (MSE) between the reconstructed and original images only on masked patches. We fixed the parameters in the encoder after the pretraining and then started to train the downstream classification task and fine‐tune the parameters of the encoder. In the downstream classification task, cotraining was adopted. We assigned different weights to the features X3, X4, and X5 extracted from the three parts of the segmentation head through the attention mechanism and then concatenated them into the new feature X (reversible process), input X into the classification head, and finally obtained the classification result. We used an SGD optimizer with a momentum of 0.9 and a weight decay of 1e‐4, the initial learning rate was 0. 01, the number of epochs was 200, and the learning rate decreased using cosine decay. Among them, the loss of the classification head was CrossEntropyloss, and the loss of the segmentation head adopted Soft‐iou‐loss and focalLoss, whose weight ratio was 1:10:10. The input images of all patients were randomly assigned to the training and test sets at the patient level. Each of our input images contained tumors. MRI images of axial T2 sequences were cropped, scaled to 256 × 256 pixels and input into the encoder. We used data augmentation (flipping and rotation) to increase the amount of data. To increase the robustness of the model, we used 5‐fold cross‐validation at the patient level and then tested the model using an independent external test set. The neural network was implemented using PyTorch 1.7, Python3.7 on an NVIDIA RTX 1080Ti GPU, Intel Xeon CPU E5‐1650, and 64G RAM.

### 2.5 Ablation analysis

To show the improvement of MAE and cotraining on the accuracy of model classification, we designed ablation experiments. We trained the following six models:

1. Model 1, which was composed of the basic framework of neural network, only performed classification tasks.
2. Model 2, which was based on Model 1 and added MAE pre‐training.
3. Model 3, which was based on Model 1 and added segmentation task, there was no information exchange between classification task and segmentation.
4. Model 4, based on Model 3, added attention mechanism to enable information exchange between segmentation task and classification task.
5. Model 5, which was based on Model 3 and added MAE pre‐training.
6. Model 6, which was based on Model 4 and added MAE pre‐training.

### 2.6 Model explanation

To demonstrate that our input images can distinguish the H3K27M genotype of the tumor, we plotted the t‐distributed stochastic neighbor embedding (t‐SNE) map. t‐SNE^29^ is a dimensionality reduction method to explore large, high‐dimensional datasets. In this study, it was used to reduce the dimensions of the high‐latitude features finally extracted from the model and visualize the features that are difficult to distinguish by the naked eye. We used saliency maps to understand which regions of the MRI image affected our deep learning model most when predicting the H3K27K alteration status. These maps can show which regions the model uses to determine the genotype of H3K27M and where the model focuses its attention.

### 2.7 Statistical analysis

We set the probability threshold for the precision calculation to 0.5. We described the performance of the classification model on each fold in terms of the accuracy, sensitivity, specificity, area under the receiver operating characteristic curve (AUROC), and area under the precision‐recall curve (AUPRC) metrics. We used Matplotlib to plot the receiver operating characteristic (ROC) curves and precision‐recall curves (PRCs). Statistical analysis was performed using SPSS software version 25.0. When *p* < 0.05, the data were considered statistically significant. We evaluated the performance of the segmentation task using the Dice coefficient, which reflects the amount of spatial overlap between the automatic segmentation masks generated by the model and the ground‐truth segmentation masks delineated by the neurosurgeon.

## 3 RESULTS

### 3.1 Characteristics of the study population

Table S2 summarizes the clinical information of all patients enrolled in the study. We included a total of 235 patients, 200 from WCHSU and 35 from CSNH. In WCHSU, 108 patients had altered H3K27M, and 92 patients had wild‐type H3K27M. The average age of the H3K27M‐altered patients was 22.05 years, which was younger than that of the wild‐type patients, which was 44.11 years. After performing a statistical analysis, a significant difference in age was observed between the two groups (22.05 ± 17.08 years vs. 44.11 ± 19.47 years, *p* < 0.000). In CSNH, 18 patients had altered H3K27M and 17 patients had wild‐type H3K27M. The average age of the H3K27M‐altered patients was 32 years, which was younger than that of the wild‐type patients (42 years), but no statistically significant difference was observed (32.72 ± 16.70 years vs. 42.82 ± 17.08 years, *p* = 0.086 > 0.05). No significant differences were detected between the sexes of patients in either dataset.

### 3.2 Deep learning model performance

In this study, the results of the model trained on the T2 sequences were better than those trained on the T1C sequences (Table S3). Therefore, we only discuss the results of the T2 sequence model below. The classification accuracy, sensitivity, and specificity of the model obtained after training are shown in Table 1. Through comparison, we found that Model 6 had the highest accuracy rate of H3K27M genotype prediction, reaching 0.905 (95% CI, 0.853–0.961). It also had the highest classification accuracy rate of 0.851 (95% CI, 0.802–0.906) in the external test set. For the baseline model, although it had a good prediction accuracy in the training set (0.855, 95% CI, 0.805–0.897), the prediction accuracy in the external test set was very low (0.697, 95% CI, 0.641–0.753). In the later model construction, we added MAE pretraining and cotraining, and the generalization performance of the model was significantly improved. Compared with Model 1, the classification accuracy of Model 2 with MAE increased by 2.5% in the training set and 3.4% in the external test set. After the MAE pretraining was added, the classification accuracy of Model 4 was improved by 3% in the training set and 3.4% in the external test set. These results show that MAE pretraining can improve the accuracy of classification prediction and the generalization performance of the model. Compared with Model 3 and Model 5, the classification accuracy of the model in the external test set after cotraining was also improved by 1.1%–2.3%, indicating that cotraining can improve the accuracy and generalization of the deep learning model for H3K27M genotype classification. In the external test set, the Dice coefficient of the Model 6 was 0.785 (95% CI, 0.759–0.811), 4.2% higher than that of Model 3.

**TABLE 1:** Diagnostic performance of each model for the prediction of H3K27M alteration.

Model	Sensitivity 95% CI		Specificity 95% CI		Accuracy 95% CI		Dice coefficient 95% CI	
	Internal	External	Internal	External	Internal	External	Internal	External
Model 1	**0.931**	**0.788**	0.777	0.611	0.855	0.697	‐	‐
	0.885–0.969	0.734–0.832	0.725–0.829	0.570–0.664	0.805–0.897	0.641–0.753		
Model 2	0.905	0.718	0.857	0.745	0.880	0.731	‐	‐
	0.851–0.953	0.657–0.776	0.809–0.911	0.693–0.787	0.825–0.943	0.672–0.791		
Model 3	0.903	0.776	0.846	0.767	0.885	0.794	0.772	0.743
	0.841–0.953	0.716–0.841	0.792–0.903	0.704–0.817	0.831–0.934	0.738–0.856	0.743–0.806	0.718–0.771
Model 4	0.843	0.706	0.903	0.922	0.875	0.817	0.783	0.759
	0.796–0.891	0.657–0.752	0.849–0.954	0.867–0.971	0.817–0.932	0.764–0.868	0.749–0.812	0.725–0.779
Model 5	0.888	0.741	0.900	0.933	0.900	0.840	0.797	0.773
	0.825–0.941	0.693–0.796	0.842–0.957	0.879–0.982	0.851–0.956	0.793–0.894	0.764–0.823	0.751–0.796
Model 6	0.867	0.764	**0.932**	**0.933**	**0.905**	**0.851**	**0.816**	**0.785**
	0.822–0.906	0.719–0.805	0.887–0.981	0.880–0.989	0.853–0.961	0.802–0.906	0.782–0.838	0.759–0.811

*Note*: Bolded values indicate that the value is the best of the six groups.

Abbreviation: CI, confidence interval.

**FIGURE 2:**
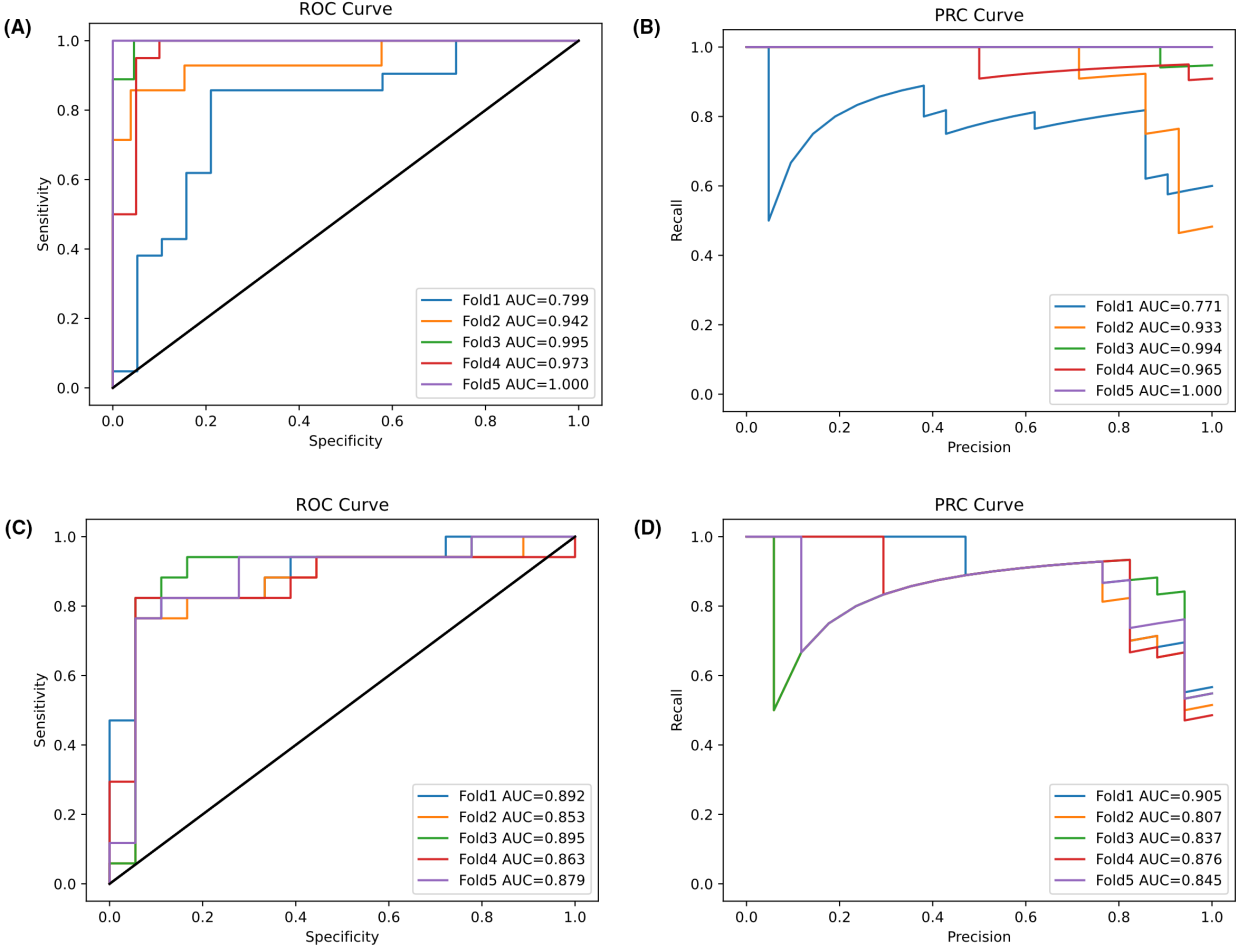
Performance of Model 6 prediction in the training set and external test set. (A) ROC analysis in the training set. (B) PRC analysis in the training set. (C) ROC analysis in the external test set. (D) PRC analysis in the external test set. Separate curves are plotted for each cross‐validation fold along with the corresponding AUC value. AUC, area under the curve.

In Table 2, we detail the results of model 6's 5‐fold cross‐validation in the training and test sets. We found that compared with the training set, the specificity in the external test set was almost not decreased (0.932 vs. 0.933), and the accuracy was slightly decreased in the external validation set (0.905 vs. 0.851). However, the accuracy of 0.851 in the external verification set was also very high (examples of incorrectly classified cases in the external set is shown in Figure S5). From Table 2, we can see not only the good prediction accuracy of the model but also the good generalization performance of the model, and generalization is an important index to evaluate the quality of the model. We plotted the ROC curve and PRC for each folded model on the training set and the external test set and calculated the area under the curve (AUC). The result is shown in Figure 2. The ROC curve was obtained on the training set, and the AUC values of folds 1–5 were 79.9%–100% and those of PRCs were 77.1%–100%. Good ROC curve results were also obtained in the external test set, with AUC values of 85.3%–89.5% for folds 1–5 and 80.7%–90.5% for PRCs.

**TABLE 2:** Cross‐validation results of Model 6 in predicting H3K27M alteration in two datasets.

Fold Number	Training set			External test set		
	Sensitivity	Specificity	Accuracy	Sensitivity	Specificity	Accuracy
Fold 1	0.810	0.842	0.825	0.765	0.944	0.857
Fold 2	0.786	0.962	0.900	0.706	0.944	0.829
Fold 3	0.889	0.955	0.925	0.823	0.944	0.886
Fold 4	0.850	0.950	0.900	0.764	0.944	0.857
Fold 5	1.000	0.952	0.975	0.764	0.889	0.829
Average	0.867	0.932	0.905	0.764	0.933	0.851

### 3.3 Data distinguishability

**FIGURE 3:**
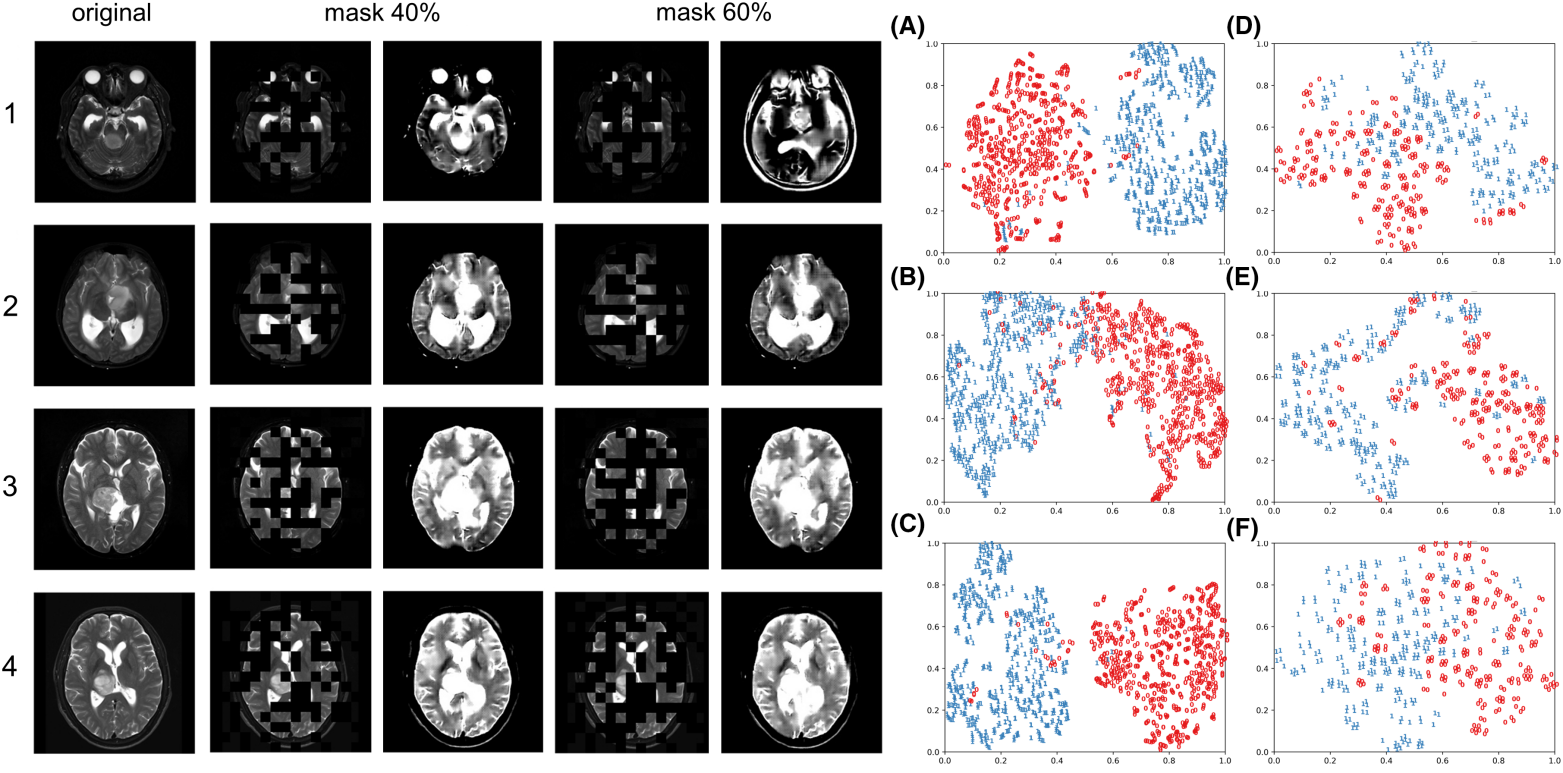
Effects of automatically reconstructed image after randomly masking 40% or 60% of the original image on the pretraining model and the t‐SNE map visualization of features. The left column shows the original image. In the middle column, the image on the right is the image reconstructed by the model using the image after 40% of random masking. In the right column, the image on the right is the image reconstructed by the model using the image after 60% of random masking. The three t‐SNE maps of A, B, and C show the differences in different H3K27M genotypes after feature extraction of the Model 1, Model 2, and Model 6 in the training set. The maps in D, E, and F show the difference after feature extraction of the above three models in the test set.

As shown in Figure 3, t‐SNE was used to visualize the contrast of features in the two datasets. Red represents the features extracted from the H3K27M‐altered group, and blue represents the features extracted from the H3K27M wild‐type group. After different model feature extraction methods were used, the image features of different H3K27M genotypes of the two groups of patients showed good distinguishability. The Model 6 had the best classification effect on the features extracted from the training set, which can almost completely distinguish patients with the two different H3K27M genotypes. The t‐SNE visualization shows that in our study, deep learning can distinguish different H3K27M genotypes using high‐dimensional information of the images of patients.

### 3.4 Visualization of the model

The original images and images after automatic reconstruction of MAE pretraining are shown in Figure 3. The MRI images of 600 normal human brains were pretrained. In the pretraining, 40% and 60% of the image blocks were randomly masked, and the whole image was automatically reconstructed using the remaining patches. MAE pretraining achieved good results in the automatic reconstruction of the masked patches, showing its ability to enhance the adaptability of the model to tasks in the head MRI scene, thus playing an important role in downstream classification tasks.

**FIGURE 4:**
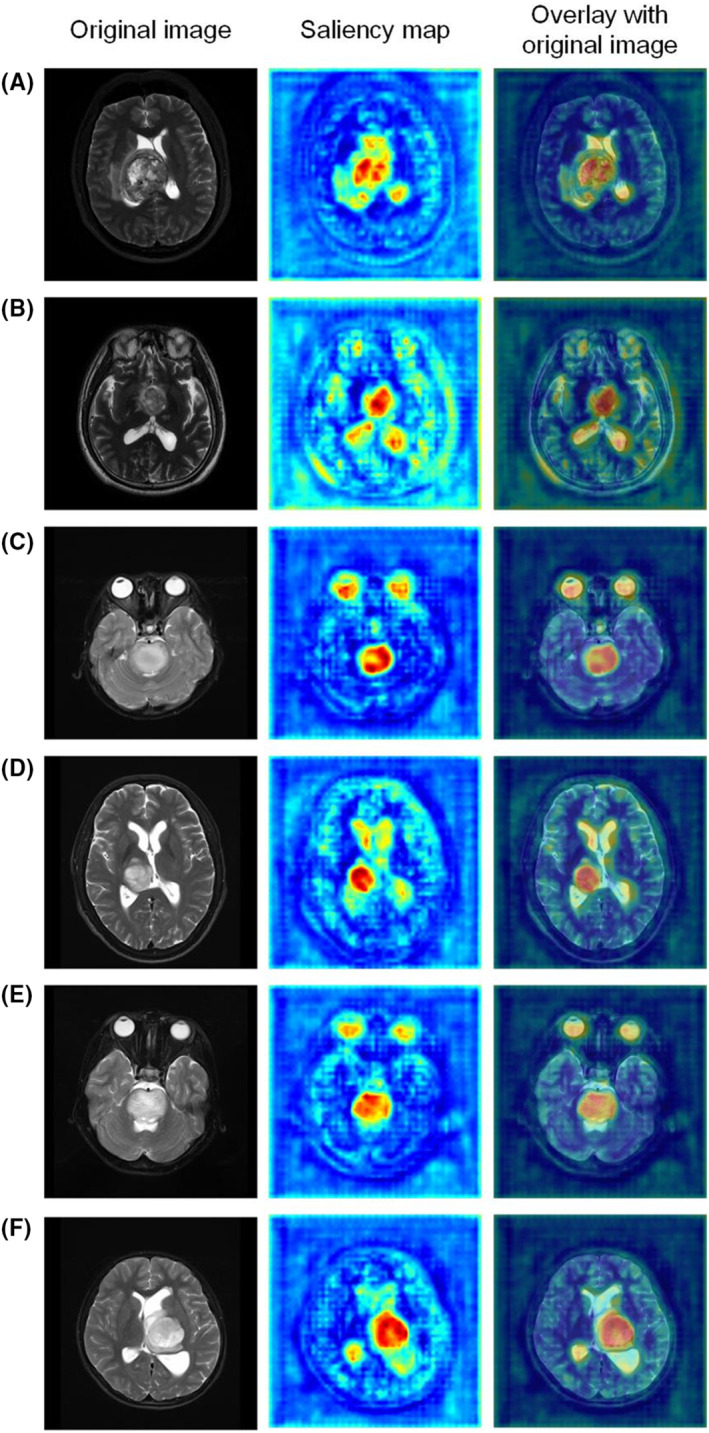
Saliency maps of the regions that Model 6 focuses on when predicting H3K27M alteration. Visual explanations of the areas of the images most important for the determination of the model prediction for H3K27M alteration. The columns are (1) the original fundus image, (2) a saliency map, and (3) a saliency map overlaying the original image.

We obtained saliency maps, as shown in Figure 4. In each map, the red and yellow regions represent the regions that are positively affected when the model predicts the H3K27M genotype. Red represents a stronger contribution than yellow, and blue areas make little contribution to prediction. We found that the central region of the tumor contributes the most to the differentiation of the H3K27M genotype, while the marginal region of the tumor and the nontumor region contribute the least to the differentiation of this genotype.

## 4 DISCUSSION

Different grades and molecular markers of gliomas have significant impacts on patient survival and treatment. The use of surgery or stereotaxic biopsy to clarify pathology is uncommon for gliomas that grow in midline locations that are surrounded by important functional tissues.^18^ Although stereotaxic brain biopsy is the gold standard for histological and genetic classification, pathological diagnoses may remain uncertain in 7%–15% of patients.^30^ In recent years, owing to the increased capacity of computing power, the wider range of data, and the availability of better models and algorithms, deep learning has developed significantly.^31^ Artificial intelligence combined with imaging omics has made remarkable achievements in medical fields, including disease classification, tumor segmentation, and target detection.^32^ In this model, MAE pretraining, a transformer module, and cotraining were added, which greatly improved the classification accuracy of the model.

At present, in medical image deep learning research, to perform a certain task, models are usually trained directly from beginning to end, which is a laborious process. Because the model is trained according to its own specific tasks, it is difficult to migrate the model to other related fields based on head MRI tasks. VGG‐16, VGG‐19, and ResNet50 networks use massive images for pretraining, and the trained models are widely used in other image recognition fields through transfer learning,^33^, ^34^, ^35^ saving considerable manpower and material resources for subsequent image recognition tasks and greatly improving the generalization performance of other models. A series of deep learning tasks based on the head MRI scene have commonality because they are all based on the head MRI scene. The pretraining model in this scene can contribute to this large category of tasks, but there is currently no pretraining model for the head MRI scene in the world. This study uses a large number of head MRI scans for pretraining, and the pretraining model could improve the accuracy and generalization performance of the H3K27M alteration recognition task. We uploaded the pretraining model and parameter weights to the internet (https://github.com/Bowen‐Huang924/MRI‐MAE‐pre‐training.git). This pretraining model can be directly applied to other deep learning tasks in the head MRI scene, and we look forward to contributing to these tasks in the future.

In recent deep learning studies related to glioma classification, classification tasks are often completed in two steps. The first step is the segmentation of the tumor region, and the second step is the classification of the tumor region after segmentation. These two steps are independent from each other, so the correlation between the two tasks is not considered.

The segmentation task was dedicated to the localization of tumor regions, while the classification task was designed to achieve the classification of the H3K27M‐mutant genotype from the representations of localized tumors. Therefore, tumor localization and characterization are closely related to the H3K27M mutation genotyping task, and the homogenous representation information of the H3K27M mutation genotype also contributes to the tumor segmentation task.^36^ Therefore, the cotraining method of the classification and segmentation tasks was adopted in this study. During the training process, the two tasks share the associated information through the information channel, Finally, the experiment proved that the effect of cotraining is better than that of single task training without information interaction.

Four articles have reported on the prediction of H3K27M molecular markers in gliomas based on artificial intelligence using radiomics. Kandemirli et al.^37^ constructed a traditional machine learning model to classify H3K27M using five image features extracted from traditional MRI sequences. They reported that the model achieved high AUC values (accuracy unreported) of 0.95 and 0.90 in the training and testing sets, respectively. Pan et al. enrolled 151 patients from a single centre, 36 selected MR image features were integrated with 3 selected clinical features to generate a model that was predictive of H3K27M mutations^25^ with an accuracy of 84.44%. In 2020, our hospital published a single‐centre machine learning model to predict H3K27M mutations. A total of 122 patients were enrolled and trained to produce 10 models, from which the best model was selected. On the validation set, the prediction accuracy of the best model was 85.5%.^38^ In this study, our model had a slightly higher prediction accuracy (90.5%) than that achieved in previous studies at our centre. Generalization ability is an important indicator for evaluating the quality of artificial intelligence models.^39^ However, none of the above studies were independently and externally validated. To evaluate the generalization ability of the proposed model, we tested it at another center and finally achieved good accuracy (85.1%). Li et al. used deep learning to predict the H3K27M mutation status in DMGs and achieved good prediction accuracies of 85.7%–90.5% in external test sets.^40^ However, they failed to account for the intrinsic relationship between segmentation and classification tasks. We removed the complicated data preprocessing steps utilized in previous studies. When predicting new cases, the data input into the model were whole‐brain images, and there was no need to manually segment the tumor on the image before inputting the image into the model, which greatly eliminated the obstacles to the clinical application of the model.

This study had two limitations. First, deep learning often requires a large amount of data training to achieve good results. We included a total of 235 patients, which is a relatively small number, but the number of cases in our center is quite large according to reports related to DMGs. At the same time, when training the model, we adopted data augmentation to expand the dataset to compensate for the data shortage to some extent. Second, we used 2D slices as the input images, which may have ignored some 3D information. However, compared with 3D models, 2D models are lighter, faster in calculation, require less computer performance, and have more cases. It is expected that this problem can be overcome in future studies with more cases.

## 5 CONCLUSION

We constructed a fully automatic deep learning model based on whole‐brain MRI to predict H3K27M alterations in DMG. The model achieved excellent accuracy in H3K27M mutation identification, and its good reproducibility and generalization performance were well supported by the external test set. The model does not require any ROI mapping or preprocessing of input images, resulting in maximum convenience for clinical application. It is expected to assist patients in noninvasive, rapid, and early diagnosis in the future.

## AUTHOR CONTRIBUTIONS

**Bowen Huang:** Data curation (equal); methodology (equal); validation (equal); writing – original draft (equal). **Yuekang Zhang:** Project administration (equal); resources (equal). **Qing Mao:** Resources (equal). **Yan Ju:** Resources (equal). **Yanhui Liu:** Resources (equal). **Zhengzheng Su:** Resources (equal). **Yinjie Lei:** Methodology (equal); software (equal); visualization (equal). **Yanming Ren:** Funding acquisition (equal); investigation (equal); writing – review and editing (equal).

## FUNDING INFORMATION

This work was supported by the Science and Technology Supportive Project of Sichuan Province (2022YFS0143, RYM; 2022YFS0049, ZYK), the Technology innovation and development project of Chengdu Science and Technology Bureau (2021‐YF05‐01038‐SN, RYM), and the Post‐Doctor Research Project, West China Hospital, Sichuan University (20HXBH033, RYM).

## CONFLICT OF INTEREST STATEMENT

The authors declare no potential conflicts of interest.

## ETHICS STATEMENT

This retrospective study was approved by Biological and Medical Ethics Committee of West China Hospital, and the requirement for informed consent was waived by Biological and Medical Ethics Committee of West China Hospital because it was a retrospective study.

## Supporting information


Figure S1.

Figure S2.

Figure S3.

Figure S4.

Figure S5.

Table S1.

Table S2.

Table S3.

## Data Availability

We uploaded the pretraining model and parameter weights to the internet (https://github.com/Bowen‐Huang924/MRI‐MAE‐pre‐training.git). The datasets used and analyzed during the current study are available from the corresponding author on reasonable request.
