# Supplementary figures and images for: Deep learning‐based prediction of H3K27M alteration in diffuse midline gliomas based on whole‐brain MRI
Source: Cancer Med. 2023 Jul 17;12(16):17139–48. doi: 10.1002/cam4.6363 (PMC10501256; doi:10.1002/cam4.6363)

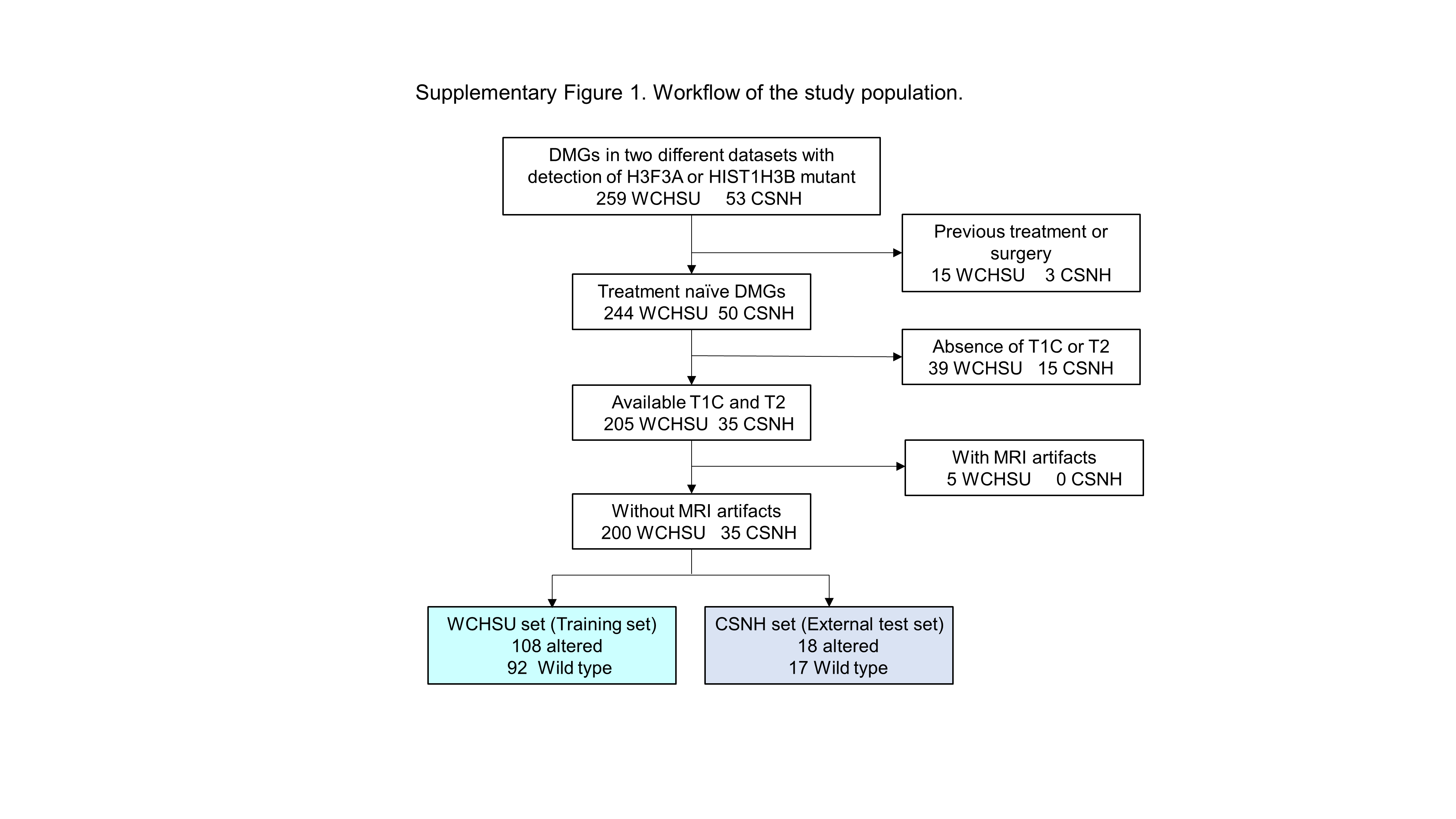

Supplement: Supplementary file 1 — Figure S1. [file CAM4-12-17139-s006.tif]

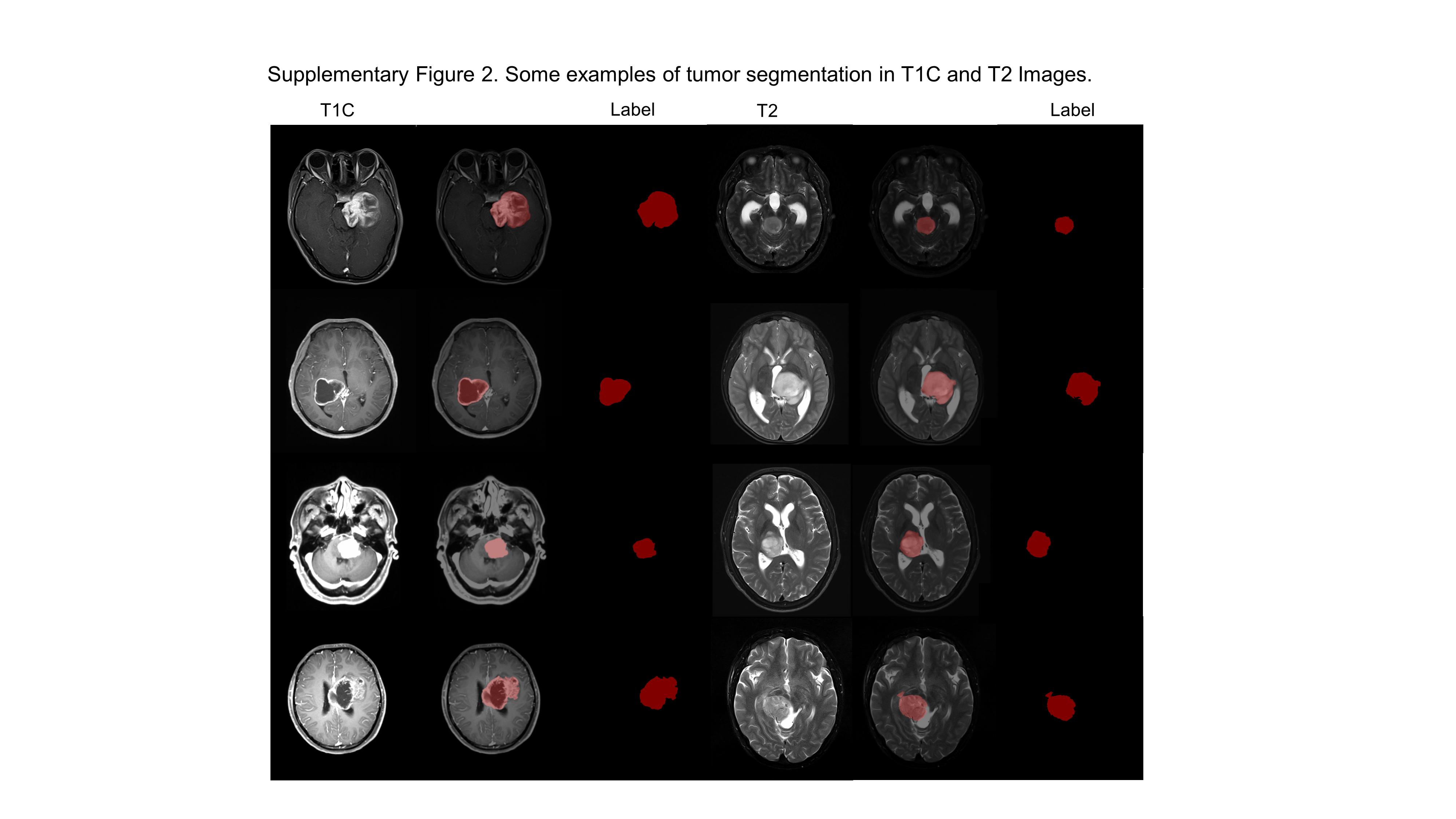

Supplement: Supplementary file 2 — Figure S2. [file CAM4-12-17139-s004.tif]

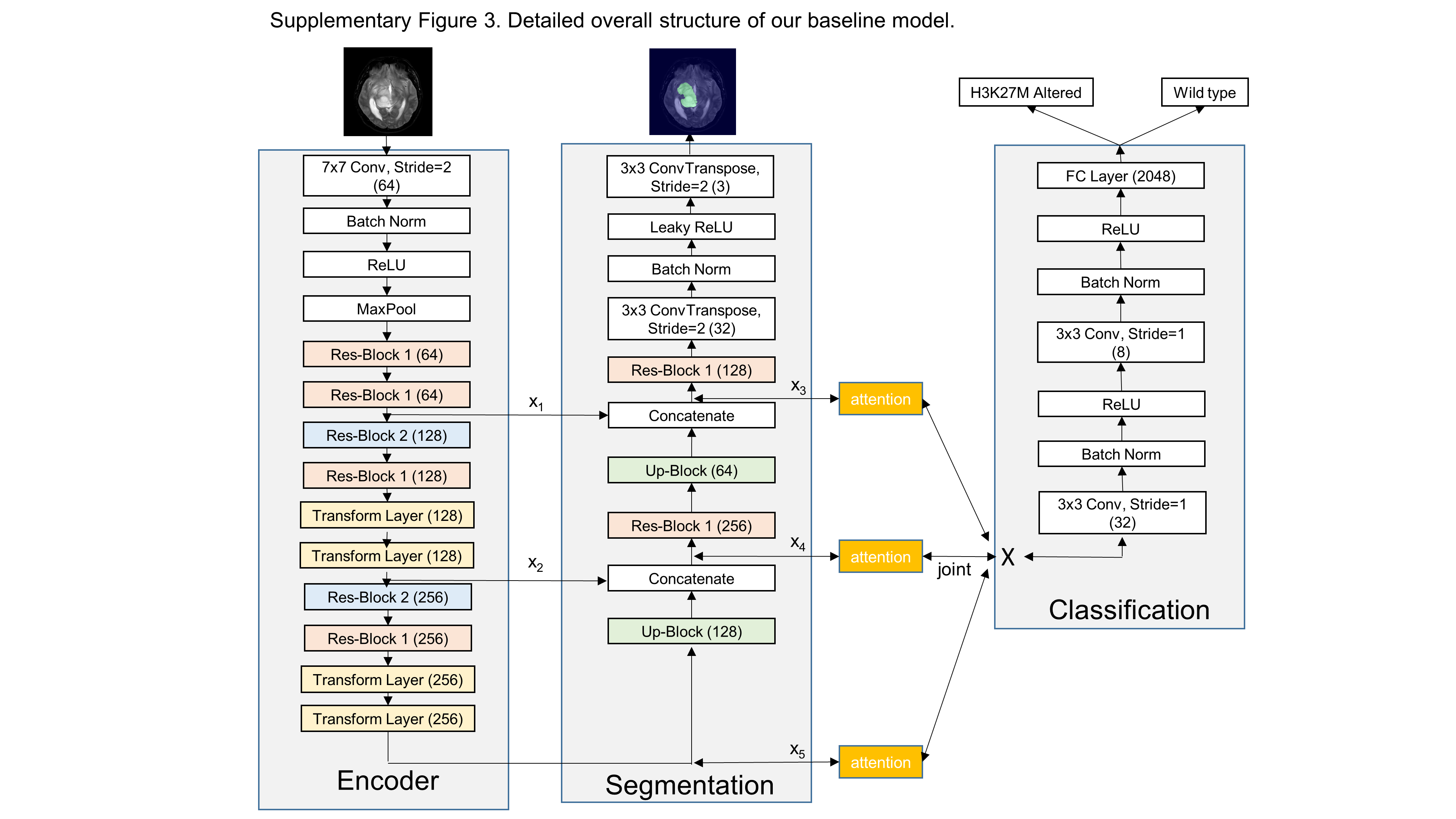

Supplement: Supplementary file 3 — Figure S3. [file CAM4-12-17139-s001.tif]

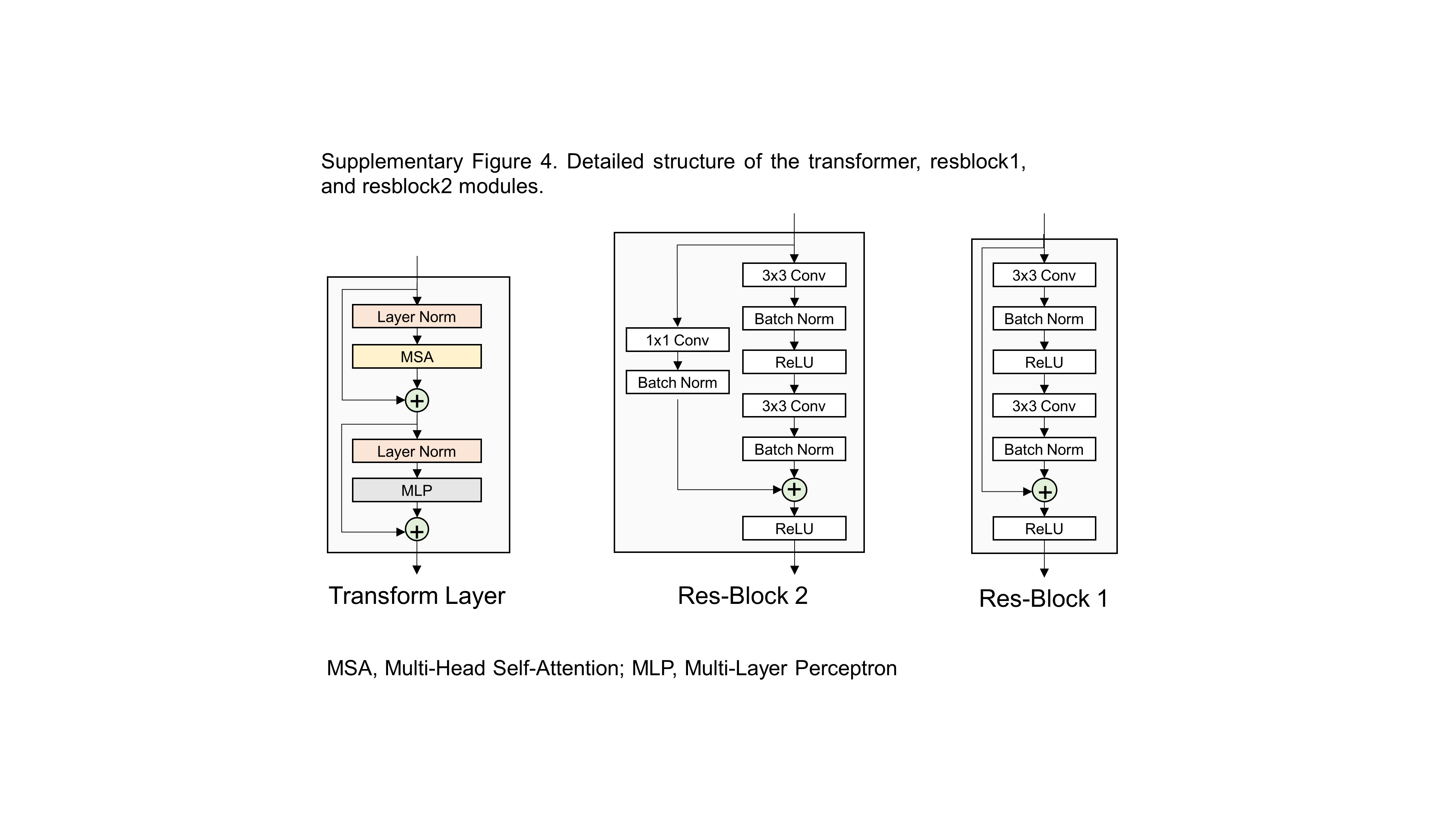

Supplement: Supplementary file 4 — Figure S4. [file CAM4-12-17139-s005.tif]

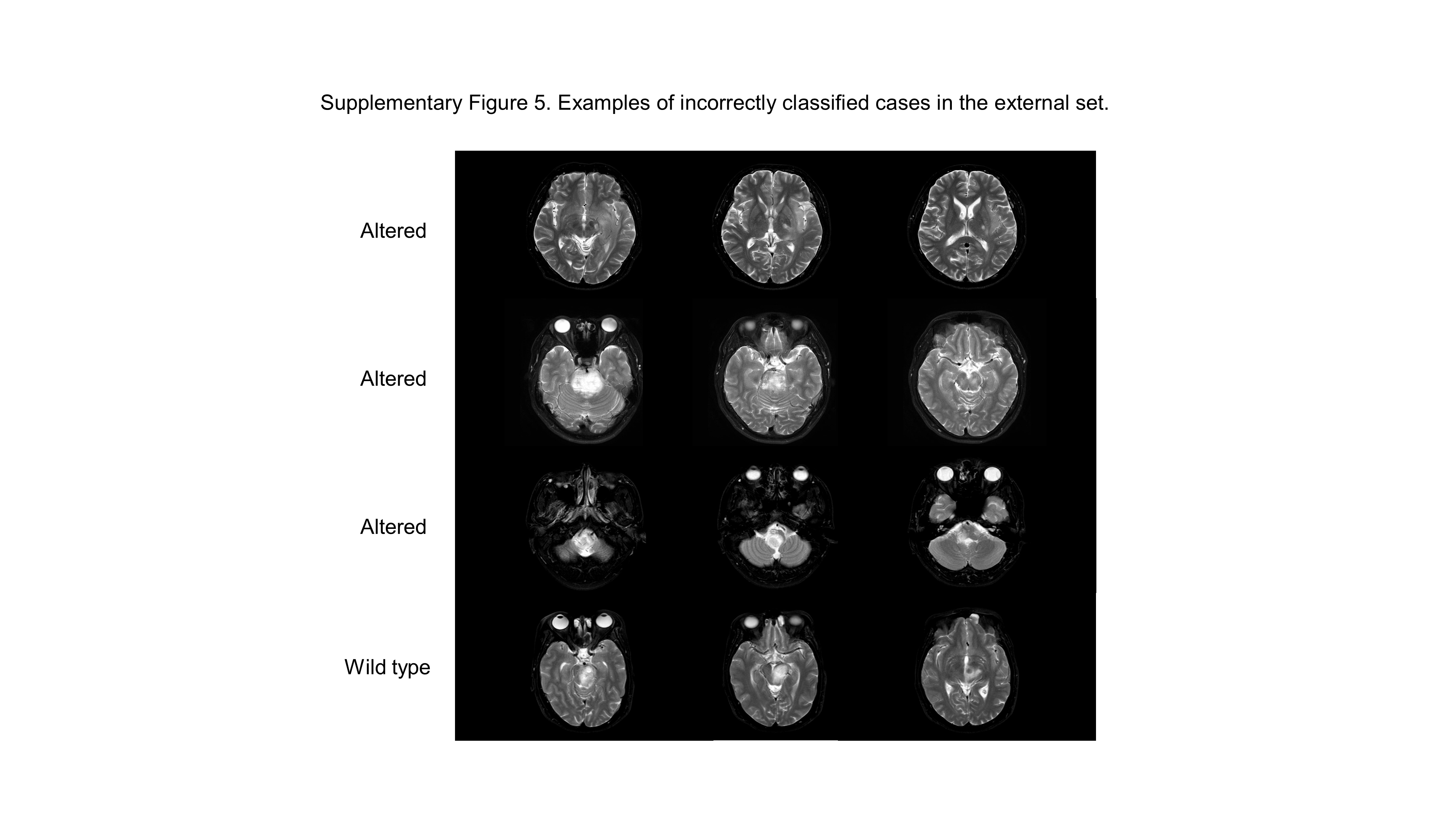

Supplement: Supplementary file 5 — Figure S5. [file CAM4-12-17139-s002.tif]
