# Supplementary material for: Deep learning‐based prediction of H3K27M alteration in diffuse midline gliomas based on whole‐brain MRI
Source: Cancer Med. 2023 Jul 17;12(16):17139–48. doi: 10.1002/cam4.6363 (PMC10501256; doi:10.1002/cam4.6363)
Supplement: Supplementary file 6 — Table S1. [file CAM4-12-17139-s003.docx]

Supplementary Table 1. Details of the most commonly used parameters for image acquisition in the WCHSU and CSNH sets

| **Image acquisition parameter** | **Parameter values (proportion)** | | | |
| --- | --- | --- | --- | --- |
|  | **WCHSU Set** | | **CSNH Set** | |
| **Magnetic field strength** | 3T(90%) | 1.5T(10%) | 3T(22.3%) | 1.5T(77.7%) |
| **Manufacturer** | SIEMENS（57.5%） | TOSHIBA_MEC  （15%） | Philips Medical Systems（77.1%） | UIH（22.9%） |
| **Model name** | TrioTim（34%） | Skyra(18.5%) | Achieva(77.1%) | uMR 588(22.9%) |
| **Spatial resolution(**mm**)** |  |  |  |  |
| T1C slice thickness | 5-5.5(49.5%) | 5.6-6(43.5%) | 5-5.5(14.3%) | 6(85.7%) |
| T1C Space between slice | 6-6.99(67.5%) | 7-8(30.5%) | 7-7.99(73.3%) | 6-6.99(14.3%) |
| T2 slice thickness | 5-5.5(55.5%) | 5.6-6(41.5%) | 5-5.5(17.1%) | 6(77.1%) |
| T2 Space between slice | 6-6.99(67.5%) | 7-8(31%) | 7-8(74.3%) | 6-6.99(11.4%) |
| **Repetition time (ms)** |  |  |  |  |
| T1 C | 1500-1700(50%) | 0-200(20.5%) | 150-160(71.4%) | 0-10(20%) |
| T2 | 2000-4500(83%) | 5000-6400(14.5%) | 3900-4000(71.4%) | 4300-4400(14.3%) |
| **Echo time (ms)** |  |  |  |  |
| T1 C | 1-3(69.5%) | 7-9(9%) | 2-2.99(77.1%) | 3-4(11.4%) |
| T2 | 100-120(41.5%) | 340-360(31.5%) | 100-109.9(77.1%) | 110-120(14.3%) |
