# Supplementary material for: Deep learning‐based prediction of H3K27M alteration in diffuse midline gliomas based on whole‐brain MRI
Source: Cancer Med. 2023 Jul 17;12(16):17139–48. doi: 10.1002/cam4.6363 (PMC10501256; doi:10.1002/cam4.6363)
Supplement: Supplementary file 7 — Table S2. [file CAM4-12-17139-s008.docx]

| Features | Training set (N=200) | | *P*-value | External test set (N=35) | | *P*-value |
| --- | --- | --- | --- | --- | --- | --- |
|  | H3K27M-altered (N=108) | H3K27M  Wild type  (N=92) |  | H3K27M-altered (N=18) | H3K27M  Wild type  (N=17) |  |
| Age (year) |  |  | <0.000 |  |  | 0.086 |
| Range | 1-74 | 2-89 |  | 10-71 | 5-60 |  |
| Mean±SD | 22.05±17.08 | 44.11±19.47 |  | 32.72±16.70 | 42.82±17.08 |  |
| Sex |  |  | 0.572 |  |  | 0.826 |
| Male | 54 | 50 |  | 11 | 11 |  |
| Female | 54 | 42 |  | 7 | 6 |  |

Supplementary Table 2. Demographics of patients with diffuse midline gliomas
