# Supplementary material for: Deep learning‐based prediction of H3K27M alteration in diffuse midline gliomas based on whole‐brain MRI
Source: Cancer Med. 2023 Jul 17;12(16):17139–48. doi: 10.1002/cam4.6363 (PMC10501256; doi:10.1002/cam4.6363)
Supplement: Supplementary file 8 — Table S3. [file CAM4-12-17139-s007.docx]

Supplementary Table 3. Diagnostic performance of each model for the prediction of H3K27M alteration on T1C sequences.

| model | Sensitivity | | Specificity | | Accuracy | |
| --- | --- | --- | --- | --- | --- | --- |
|  | Internal | External | Internal | External | Internal | External |
| Model 1 | 0.815 | 0.692 | 0.733 | 0.594 | 0.737 | 0.634 |
| Model 2 | 0.802 | 0.671 | 0.729 | 0.625 | 0.762 | 0.645 |
| Model 3 | 0.801 | 0.685 | 0.756 | 0.663 | 0.778 | 0.671 |
| Model 4 | 0.808 | 0.684 | 0.781 | 0.752 | 0.779 | 0.690 |
| Model 5 | 0.816 | 0.692 | 0.792 | 0.763 | 0.796 | 0.711 |
| Model 6 | **0.827** | **0.719** | **0.815** | **0.770** | **0.811** | **0.734** |
